# Pattern and Predictive Factors of Metastasis in Lymph Nodes Posterior to the Right Recurrent Laryngeal Nerve in Papillary Thyroid Carcinoma

**DOI:** 10.3389/fendo.2022.914946

**Published:** 2022-07-18

**Authors:** Mengqian Zhou, Yuansheng Duan, Beibei Ye, Yuxuan Wang, Hong Li, Yue Wu, Peng Chen, Jiajia Zhu, Chao Jing, Yansheng Wu, Xudong Wang

**Affiliations:** Department of Otorhinolaryngology and Maxillofacial Oncology, Tianjin Medical University Cancer Institute and Hospital, National Clinical Research Center for Cancer, Tianjin’s Key Laboratory of Cancer Prevention and Therapy, Tianjin’s Clinical Research Center for Cancer, Tianjin, China

**Keywords:** papillary thyroid carcinoma, central lymph node metastasis, recurrent laryngeal nerve, extrathyroidal extension, risk factors

## Abstract

**Objective:**

The right cervical central lymph nodes include lymph nodes anterior to the right recurrent laryngeal nerve (LN-arRLN) and lymph nodes posterior to the right recurrent laryngeal nerve (LN-prRLN), and are separated by the right recurrent laryngeal nerve (RLN). LN-prRLN is a common site of nodal recurrence after the resection of papillary thyroid carcinoma (PTC). However, the complexity in anatomical structure brings difficulties in determining the surgical scope, so it is necessary to assess the pattern and predictive factors of right cervical central lymph nodes, especially LN-prRLN metastasis in papillary thyroid carcinoma.

**Methods:**

A total of 562 diagnosed PTC patients who underwent right or total thyroidectomy were enrolled in this retrospective study. The clinicopathological features were collected, univariate and multivariate analyses were performed to determine predictive factors of the right central lymph node metastasis.

**Results:**

In this study, the metastatic rates of the right CLN, the LN-arRLN and the LN-prRLN were 59.6% (335/562), 51.8% (291/562) and 30.4% (171/562), respectively. And 22.6% (127/562) of patients had both LN-arRLN and LN-prRLN metastasis. Among patients without LN-arRLN metastasis, the rate of LN-prRLN metastasis was 16.2% (44/271), accounting for 25.7% of the LN-prRLN metastasis group. Factors associated with an increased risk of LN-arRLN metastasis include male, age below 55 years, tumor size > 1cm, extrathyroidal extension (ETE), clinical lymph nodes metastasis(cN1), lateral lymph node metastasis, and left CLN metastasis. In addition, ETE, lateral lymph node metastasis, and LN-arRLN metastasis were independent factors of LN-prRLN metastasis. The predictive factors of LN-prRLN in cN0 PTC were further explored, revealing that tumor size ≥1.5cm, ETE, and LN-arRLN metastasis were independent predictors of LN-prRLN metastasis in cN0 PTC.

**Conclusion:**

The LN-prRLN should not be ignored in surgery because of its high rate of metastasis. Our findings indicate that thorough dissection of central lymph nodes, especially LN-prRLN is crucial in clinical work.

## Introduction

Thyroid papillary carcinoma (PTC) is one of the most common histological types of thyroid cancer, with an increasing occurrence and high lymph node metastasis rates (1). PTC treatment mainly includes thyroidectomy and lymph node dissection. Research has shown that PTC lymph node metastasis incidence is 30% to 90%, with central lymph node metastasis being the most common site (2).

The left and right sides of the central cervical lymph nodes are symmetrical, but there are some differences in the anatomy. The left recurrent laryngeal nerve is located next to the esophagus, and the right recurrent laryngeal nerve ascends through the adipose tissue. The right central neck lymph nodes are divided into the anterior part (LN-arRLN) and posterior part (LN-prRLN) (3). The LN-prRLN extends superiorly to the hyoideum, inferiorly to the rostral border of the manubrium, anteriorly to the right recurrent laryngeal nerve, and the posterior border is the anterior wall of the airway, including the space between the nerve and the tracheoesophageal groove, filled by soft tissues and lymphoid tissues. These structures are obviously different from the left central cervical lymph nodes. Domestic and foreign guidelines explain the necessity of lymph node dissection in the central region of thyroid cancer. Thorough lymph node dissection is the foundation of the therapeutic effect.

The American Thyroid Association (ATA) guidelines outline the anatomical boundaries of central lymph node dissection (CLND) in thyroid cancer (4), which only define the width of the central compartment and not the depth of the central compartment (5). Whether the lymph node of LN-prRLN should be dissected is still controversial. On the one hand, some researchers suggest that there is no need for routine LN-prRLN lymph node dissection because of the low metastasis rate, the complex surgical approach and the high complication rate as the LN-prRLN is located in a deep position with narrow exposure. The preoperative assessment of metastasis is challenging. This area would likely be overlooked even by experienced surgeons. Furthermore, dissection of the lymphatic and adjacent adipose tissues in the area posterior to the RLN may cause various complications, such as nerve injury, parathyroid gland damage and chylous fistula. On the other hand, some scholars have pointed out that the LN-prRLN metastasis rate in PTC patients is as high as 2.74–38.27% (6, 7). The initial surgical treatment plan for LN-prRLN is very important for the patient’s prognosis. Clayman et al. reported 60% of recurrent central neck nodes were found dorsal to the RLN (8). Furthermore, incomplete CLN dissection may result in increased risks of recurrence and complications due to multiple operations. If LN-prRLN lymph nodes are not thoroughly dissected, reoperation would be very challenging and some patients may lose the chance of radical cure (7). It is possible that the remaining lymph nodes eventually lead to recurrence. Some researchers suggested routinely clearing LN-prRLN to optimize the treatment outcome, as the dissection of cervical LNs during initial operation is essential for patient prognosis (9). These points of contention prove that LN-prRLN deserves attention and further research. Therefore, it is urgent to assess the risk of LN-prRLN metastasis accurately, which could help to determine the scope of surgery and reduce recurrence and mortality.

In order to provide a basis for formulating standardized treatments, this study retrospectively analyzed the clinical data of 562 patients with PTC and explored the related factors that affect the metastasis of LN-prRLN.

## Materials and Methods

### Patients

This single-center retrospective cohort study consisted of patients from Tianjin Medical University Cancer Institute & Hospital between January 2015 and January 2021. The inclusion criteria were:(1) patients with a pathological diagnosis of PTC; (2) tumor lesions located in the right or bilateral thyroid gland; (3) all patients underwent lymph node dissection, including at least the right central lymph nodes; (4) without a history of previous carcinoma or thyroidectomy.

### Pathological Examination

Postoperative pathological examinations of all patients were diagnosed by two experienced pathologists according to WHO guidelines. The central lymph nodes were examined separately to investigate metastatic involvement.

### Statistical Analyses

All statistical analyses were performed using the SPSS 23.0 software (Chicago, IL, USA). Univariate analysis for the comparison between patient groups was performed with Pearson’s chi-square test or Fisher’s exact test. The significant factors from the univariate analysis were incorporated into the multivariate analysis. The Cox proportional hazards model was constructed to identify predictive factors of LN-arRLN and LN-prRLN metastasis. A p-value < 0.05 was considered statistically significant.

## Results

### Baseline Data of Enrolled Patients

The base clinicopathological data of enrolled patients are summarized in **Table 1**. Among 562 patients, 21.4% (120/562) were male and 78.6% (442/562) were female patients. The mean age was 43.5 ± 11.3 years. The mean tumor size was 1.37 ± 0.79cm (range 0.3–7.1 cm). In total, 37.2% (209/562) patients had lesions with uni-multifocality (unilateral multifocality), 39.1% (220/562) had extrathyroidal extension, and 13.0% had prelaryngeal lymph nodes metastasis. Histopathological examination showed that 125 (22.2%) patients had coexistent Hashimoto’s thyroiditis in this study. Tumors located in the upper portion of the gland were detected in 164 (29.2%) patients, and tumors in the middle and lower lobe of the thyroid were detected in 91(16.2%) and 270 (48%) patients, respectively. Diffuse lesions were observed in 37(6.6%) patients. Clinical occult lymph nodes (cN0) were present in 325 (57.7%) patients.

**Table 1:** Baseline data of 562 PTC patients.

Features	N=562 (%)
Gender	
Male	120 (21.4)
Female	442 (78.6)
Age (mean, range)	43.5 ± 11.3
Mean tumor size ± SD (range), cm	1.37 ± 0.79 (0.3-7.1)
Uni-multifocality	
No	353 (62.8)
Yes	209 (37.2)
lesion location	
Upper	164 (29.2)
Middle	91 (16.2)
Lower	270 (48)
Diffuse	37 (6.6)
ETE	
No	342 (60.9)
Yes	220 (39.1)
clinical N classification	
cN0	325 (57.7)
cN1	238 (42.3)
Prelaryngeal lymph nodes metastasis	
No	489 (87.0)
Yes	73 (13.0)
Coexistent Hashimoto’s thyroiditis	125 (22.2)

### Pattern of the Right Central Lymph Node Metastasis in 562 Patients

Positive right CLN was observed in 335 (59.6%) patients, and the rates of LN-arRLN and LN-prRLN metastasis were 51.8% (291/562) and 30.4% (171/562), respectively. 22.6% (127/562) patients had both LN-arRLN and LN-prRLN metastasis. In addition, among patients without LN-arRLN metastasis, the rate of LN-prRLN metastasis was 16.2% (44/271), as displayed in **Table 2**.

**Table 2:** Pattern of the right central lymph node metastasis in 562 patients.

Metastatic pattern		LN-arRLN metastasis	
		Positive (n=291)	Negative (n=271)
LN-prRLN	Positive (n=171)	127 (22.6)	44 (7.8)
	Negative (n=391)	164 (29.2)	227 (40.4)

### Predictors of LN-arRLN Metastasis

Univariate analysis showed that LN-arRLN metastasis was significantly associated with gender (male), age (<55 years), tumor size (>1 cm), uni-multifocality, ETE, clinical N classification (cN1), prelaryngeal lymph nodes metastasis, right lateral lymph node metastasis and left CLN metastasis. However, tumor location and coexistent Hashimoto thyroiditis were not significantly associated with LN-arRLN metastasis (P > 0.05) (**Table 3**). The COX regression analysis, including the significant predictors identified in the univariate analysis, was performed to determine the independent factors associated with LN-arRLN metastasis following adjustment for various other factors. As shown in **Table 4**, significant associations were observed between LN-arRLN metastasis and the following tumor characteristics: gender, age, tumor size, ETE, clinical N classification, right lateral lymph node metastasis and left CLN metastasis.

**Table 3:** Univariate analysis of predictor for lymph nodes anterior to the right recurrent laryngeal nerve (LN-arRLN).

Features	LN-arRLN metastasis		χ2	P
	Positive (n=291)	Negative (n=271)		
Gender				
Male	82 (68.3)	38 (31.7)	16.746	0.000
Female	209 (47.3)	233 (52.7)		
Age				
<55	257 (56.9)	195 (43.1)	23.859	0.000
≥55	34 (30.9)	76 (69.1)		
Tumor size (cm)				
≤1	85 (37.4)	142 (62.6)	31.339	0.000
>1	206 (61.5)	129 (38.5)		
Uni-multifocality				
No	169 (47.9)	184 (52.1)	5.794	0.016
Yes	122 (58.4)	87 (41.6)		
lesion location				
Upper	95 (57.9)	69 (42.1)	6.087	0.107
Middle	40 (44.0)	51 (56.0)		
Lower	134 (49.6)	136 (50.4)		
Diffuse	22 (59.5)	15 (40.5)		
ETE				
No	113 (33.0)	229 (67.0)	122.862	0.000
Yes	178 (80.9)	42 (19.1)		
Clinical N classification				
cN0	148 (45.7)	176 (54.3)	11.403	0.001
cN1	143 (60.1)	95 (39.9)		
Prelaryngeal lymph nodes metastasis				
No	229 (46.8)	260 (53.2)	36.930	0.000
Yes	62 (84.9)	11 (15.1)		
Coexistent Hashimoto’s thyroiditis				
No	226 (51.7)	211 (48.3)	0.003	0.955
Yes	65 (52.0)	60 (48.0)		
Lateral lymph node metastasis				
No	182 (41.5)	257 (58.5)	85.584	0.000
Yes	109 (88.6)	14 (11.4)		
Left CLN metastasis				
No	150 (39.4)	231 (60.6)	72.960	0.000
Yes	141 (77.9)	40 (22.1)		

**Table 4:** Multivariate Analysis of clinicopathologic characteristics in LN-arRLN lymph node metastasis.

	B	SE	Wals	Sig.	Exp (B)	Exp (B)95% Confidence Interval
Gender	-0.600	0.273	4.820	0.028	0.549	0.321-0.938
Age	-0.903	0.272	11.016	0.001	0.405	0.238-0.691
Tumor size(cm)	0.564	0.220	6.580	0.010	1.757	1.142-2.703
Uni-multifocality	-0.127	0.231	0.305	0.581	0.880	0.560-1.384
ETE	1.625	0.228	50.613	0.000	5.079	3.246-7.948
Clinical N classification	0.480	0.216	4.932	0.026	1.616	1.058-2.469
Prelaryngeal lymph nodes metastasis	0.677	0.417	2.637	0.104	1.968	0.869-4.454
Lateral lymph node metastasis	1.482	0.341	18.940	0.000	4.402	2.258-8.581
Left CLN metastasis	0.773	0.262	8.671	0.003	2.166	1.295-3.622
Constant	-0.273	0.530	0.265	0.607	0.761	

### Predictors of LN-prRLN Metastasis

The univariate analysis identified gender, age, tumor size, uni-multifocality, ETE, prelaryngeal lymph nodes metastasis, right lateral lymph node metastasis, left CLN metastasis and LN-arRLN metastasis as predictors of LN-prRLN metastasis (**Table 5**). In the corresponding multivariate analysis, the results demonstrated that ETE, lateral lymph node metastasis, and the number of metastatic LN-arRLN were associated with LN-prRLN metastasis (**Table 6**). As shown in the table, the rate of LN-prRLN metastasis in the LN-arRLN metastasis group was significantly higher than that of the non-metastasis group.

**Table 5:** Univariate analysis of predictor for lymph node posterior to the right recurrent laryngeal nerve (LN-prRLN).

Features	LN-prRLN metastasis		χ2	P
Positive (n=171)		Negative (n=391)
Gender				
Male	49 (40.8)	71 (59.2)	7.805	0.005
Female	122 (27.6)	320 (72.4)		
Age				
<55	151 (33.4)	301 (66.6)	9.688	0.002
≥55	20 (18.2)	90 (81.8)		
Tumor size (cm)				
≤1	54 (23.8)	173 (76.2)	7.928	0.005
>1	117 (34.9)	218 (65.1)		
Uni-multifocality				
No	96 (27.2)	257 (72.8)	4.683	0.030
Yes	75 (35.9)	134 (64.1)		
lesion location				
Upper	49 (29.9)	115 (70.1)	3.834	0.280
Middle	30 (33.0)	61 (67.0)		
Lower	76 (28.1)	194 (71.9)		
Diffuse	16 (43.2)	21 (56.8)		
ETE				
No	30 (8.8)	312 (91.2)	193.535	0.000
Yes	141 (64.1)	79 (35.9)		
Clinical N classification				
cN0	95 (29.3)	229 (70.7)	0.442	0.506
cN1	76 (31.9)	162 (68.1)		
Prelaryngeal lymph nodes metastasis				
No	136 (27.8)	353 (72.2)	12.163	0.000
Yes	35 (47.9)	38 (52.1)		
Coexistent Hashimoto’s thyroiditis				
No	141 (32.3)	296 (67.7)	3.137	0.077
Yes	30 (24.0)	95 (76.0)		
Lateral lymph node metastasis				
No	94 (21.4)	345 (78.6)	77.002	0.000
Yes	77 (62.6)	46 (37.4)		
Left CLN metastasis				
No	85 (22.3)	296 (77.7)	36.822	0.000
Yes	86 (47.5)	95 (52.5)		
LN-arRLN metastasis				
0	44 (16.2)	227 (83.8)	56.802	0.000
1	33 (33.7)	65 (66.3)		
2	32 (50.0)	32 (50.0)		
≥3	62 (48.1)	67 (51.9)		

**Table 6:** Multivariate analysis of clinicopathologic characteristics in LN-prRLN lymph node metastasis.

	B	SE	Wals	Sig.	Exp (B)	Exp (B) 95% Confidence Interval
Gender	-0.208	0.283	0.538	0.463	0.812	0.467-1.415
Age	-0.349	0.330	1.119	0.290	0.705	0.369-1.347
Tumor size (cm)	-0.144	0.265	0.294	0.588	0.866	0.515-1.457
Uni-multifocality	-0.010	0.260	0.001	0.970	0.990	0.595-1.648
ETE	3.000	0.295	103.383	0.000	20.076	11.261-35.792
Prelaryngeal lymph nodes metastasis	-0.373	0.352	1.124	0.289	0.689	0.346-1.372
Lateral lymph node metastasis	1.144	0.289	15.691	0.000	3.140	1.783-5.532
Left CLN metastasis	0.439	0.290	2.295	0.130	1.552	0.879-2.74
Number of LN-arRLN metastasis			9.128	0.028		
1	0.789	0.359	4.817	0.028	2.200	1.088-4.449
2	1.049	0.389	7.289	0.007	2.855	1.333-6.115
≥3	0.803	0.388	4.280	0.039	2.232	1.043-4.774
Constant	-2.877	0.667	18.624	0.000	0.056	

B, Beta coefficient; SE, Standard error of the mean; Sig, Statistical significance; CI, Confidence interval.

### Logistic Regression Model to Predict Risk Factors for LN-prRLN Metastasis

**Figure 1:**
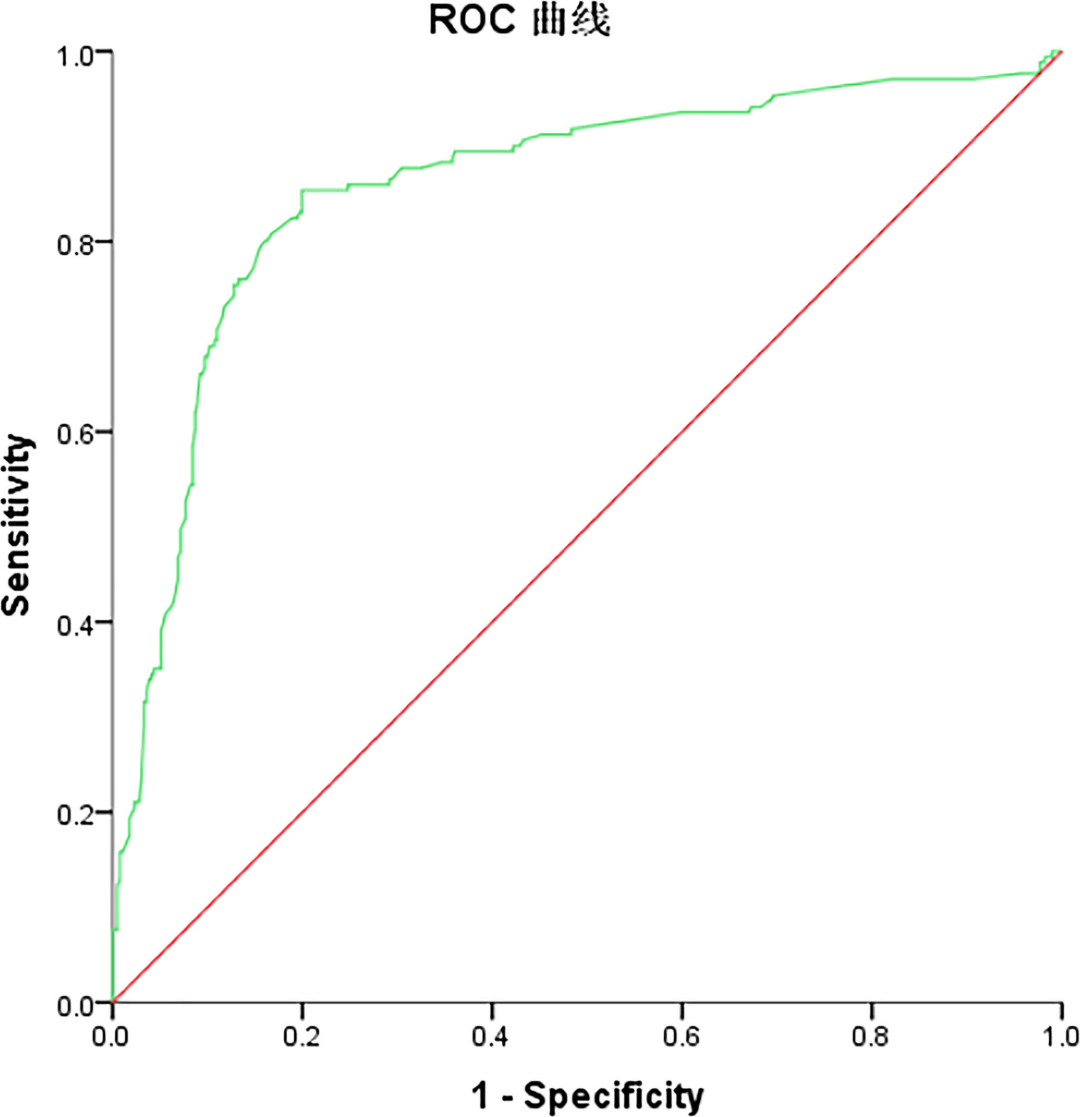
ROC curve of the logistic regression model for predicting LN-prRLN metastasis.

A logistic regression model was established based on the results of the multivariate analysis: logistic (P)= -2.877- 0.208 × gender -0.349 × age -0.144 × tumor size -0.010 × Uni-multifocality +3.000 ×ETE - 0.373 ×prelaryngeal lymph nodes metastasis + 1.144 × lateral lymph node metastasis +0.439 × left CLN metastasis+0.789/1.049/0.803 × number of LN-arRLN metastasis. The values of each factor were substituted into the equation to obtain the probability of multi-factor prediction, and the ROC curve was calculated (**Figure 1**). The predictive model comprehensively reflects the predictive value of the five risk factors. The area under the curve (AUC) of the model for predicting LN-prRLN metastasis was 0.857 (95% CI, 0.821–0.894), the cut-off value was 0.284, with a sensitivity of 85.4% and a specificity of 80.1%. The maximum Youden index was 0.655.

### Risk Factors for Predicting LN-prRLN in cN0 PTC

In this study, we analyzed the clinical lymph node status (clinical N classification) and found that cN1 was closely related with LN-arRLN metastasis (p= 0.026), but the clinical N classification had no association with LN-prRLN metastasis. As controversy still exists regarding the dissection of LN-prRLN in cN0 PTC, further analysis of the predictors of LN-prRLN metastasis in these patients was carried out. As shown in **Table 7**, an increased risk of LN-prRLN metastasis was observed in tumors larger than 1.5cm, tumors with extrathyroidal extension, left CLN metastasis, or LN-arRLN metastasis. The multivariate analysis identified the tumor size, ETE and LN-arRLN metastasis as independent factors predicting LN-prRLN metastasis (**Table 8**).

**Table 7:** Univariate analysis of predictor for LN-prRLN in cN0 PTC.

Features	LN-prRLN metastasis		χ2	P
	Positive (n=54)	Negative (n=270)		
Gender				
Male	15 (24.6)	46 (75.4)	3.397	0.065
Female	39 (14.8)	224 (85.2)		
Age				
<55	44 (17.8)	203 (82.2)	0.985	0.321
≥55	10 (13.0)	67 (87.0)		
Tumor size (cm)				
≤1.5	36 (13.6)	229 (86.4)	9.951	0.002
>1.5	18 (30.5)	41 (69.5)		
Uni-multifocality				
No	38 (16.8)	188 (83.2)	0.012	0.914
Yes	16 (16.3)	82 (83.7)		
lesion location				
Upper	15 (16.1)	78 (83.9)	0.092	0.955
Middle	9 (15.8)	48 (82.4)		
Lower	30 (17.2)	144 (82.8)		
ETE				
No	11 (4.5)	233 (95.5)	105.180	0.000
Yes	43 (53.8)	37 (46.3)		
Left CLN metastasis				
No	40 (14.7)	232 (85.3)	4.691	0.030
Yes	14 (26.9)	38 (73.1)		
LN-arRLN metastasis				
No	28 (13.1)	185 (86.9)	5.550	0.018
Yes	26 (23.4)	85 (76.6)		

**Table 8:** Multivariate analysis of predictor for LN-prRLN in cN0 PTC.

	B	SE	Wals	Sig.	Exp (B)	Exp (B) 95% Confidence Interval
Tumor size (cm)	0.382	0.150	6.513	0.011	1.465	1.093-1.965
ETE	3.784	0.486	60.630	0.000	43.988	16.97-114.021
Left CLN metastasis	-0.126	0.488	0.067	0.796	0.882	0.339-2.296
LN-arRLN metastasis	1.070	0.467	5.248	0.022	2.915	1.167-7.279
Constant	-4.582	0.611	56.236	0.000	0.010	

B, Beta coefficient; SE, Standard error of the mean; Sig, Statistical significance; CI, Confidence interval.

## Discussion

The guidelines of the American Thyroid Association recommend the use of preoperative ultrasonography to clinically detect relevant nodal disease and support the management of central lymph node dissection (4). Cervical lymph node metastasis is diagnosed in 20% to 90% of PTC patients, and CLN metastasis is significantly associated with recurrence (10). Therefore, thorough dissection of the central area during the initial operation is crucial. Due to the difference in the anatomical structures of the left and right central area, there is no absolute uniform standard defining the integrity of the right central area.

Whether to dissect the LN-prRLN posterior to the recurrent laryngeal nerve in the absence of definite positive lymph nodes is currently debated. Some scholars believe that PTC is a kind of low-grade tumor, and it is unnecessary to perform LN-prRLN dissection, exposing the patient to surgical risks and affecting the postoperative quality of life. However, other scholars believe that although PTC has a good prognosis, the rate of lymph node metastasis is high, and the central lymph nodes are the most common metastatic sites. Thorough initial dissection is crucial to avoid local recurrence and metastasis. Simultaneous dissection in LN-prRLN could reduce the recurrence in the central area (4), reduce medical costs, and avoid the high complication rates caused by secondary surgery. The main reasons for the controversy around CCND include the balance of the potential benefits opposed to a higher incidence of postoperative complications when LN-prRLN dissection is performed. Although LN-prRLN is located within the central compartment, they are frequently not removed during thyroid surgery due to the high risk of injury to the right RLN and parathyroid gland. Some researchers reported that extensive LN dissection might increase morbidity without benefiting surgical outcomes and survival rates (11). Thus, it is important to identify the risk factors associated with LN-prRLN metastasis in PTC, which could assist surgeons in deciding whether to perform LN-prRLN dissection. In addition, little has been reported about predictors of LN-prRLN metastasis in patients with PTC.

From previous studies, the rate of LN-arRLN metastasis is about 8.65-19.8% (12–14). In this study, LN-arRLN metastasis was observed in 51.8% (291 of 562) of patients, while LN-prRLN metastasis was found in 30.4% (171 of 562). This difference in incidence rates could be related to the limited number of cases in this study. This study did not find a significant association between gender, age, tumor size, uni-multifocality, prelaryngeal lymph nodes metastasis, left CLN metastasis, and LN-prRLN metastasis. However, a significant association was reported by previous studies on PTC (14–17). Using univariate and multivariate analyses, we confirmed that ETE, lateral lymph node metastasis, the number of metastatic LN-arRLN were independent predictors of LN-prRLN metastasis, which was consistent with previous reports (6, 14, 15, 18).

Although multifocality was previously found to be associated with LN-prRLN metastasis (19, 20), our findings suggested that multifocality and tumor location appeared to be non-significant risk factors of LN-prRLN metastasis in PTC patients. However, tumor size was closely associated with increased odds of LN-arRLN metastasis rather than LN-prRLN metastasis. Some studies have reported that LN-arRLN metastasis is positively related to primary tumor size (21). The results demonstrated that the cut-off value of the tumor size in PTC for increased LN-arRLN metastasis risk was 1cm. Therefore, we consider that a tumor size >1cm could be the threshold for increased risk of LN-arRLN metastasis.

In several studies, LN-arRLN metastasis has been proven to be an important factor for LN-prRLN metastasis in PTC (6, 14). In this study, the risk of LN-prRLN metastasis was almost twice higher in patients with LN-arRLN metastasis than those without, indicating that the LN-arRLN compartment could serve as an intraoperative factor for predicting LN-prRLN metastasis. Careful examination of the LN-prRLN compartment should be performed with clinical or pathological LN-arRLN metastasis in PTC patients. Previous retrospective studies argue that lateral lymph nodes metastasis is an independent risk factor of LN-prRLN metastasis (22, 23). The role of ETE in predicting LN-prRLN metastasis also has been supported by some research (24, 25). Our results imply that in patients with ETE, lateral lymph nodes metastasis, or LN-arRLN metastasis, the LN-prRLN should be removed with complete neck dissection to prevent metastasis.

As increasing evidence shows a relatively high rate of clinically occult central neck lymph node metastasis ranging from 25.7% to 60% (26), LN-prRLN metastasis could occur in up to 26.6% in cN0 PTC patients (27–29). Some investigations indicated that additional removal procedures are not associated with increased risk of transient or permanent hypoparathyroidism and vocal cord paralysis (30). Resection in cN0 patients is still controversial. Some scholars pointed out that resection is still the best choice for clinical cN0 patients (26, 29). Furthermore, the resection of the lymph nodes posterior to the right recurrent laryngeal nerve is often overlooked, resulting in postoperative recurrence. Therefore, we further analyzed relative factors associated with LN-prRLN metastasis in cN0 patients. Based on univariate and multivariate analysis, the results revealed that tumor size exceeding 1.5cm, ETE and LN-arRLN metastasis were independent predictors of LN-prRLN in these patients.

Some researchers suggest that detecting LN-prRLN during the initial operation is very important to reduce the reoperation rate and decrease associated complications (31). However, LN-prRLN dissection is not routinely performed. The reason is that the metastasis rate in this area is not high, but the surgical technique requirement is high, which may lead to postoperative complications. However, if LN-prRLN dissection is not performed, surgical resection after the recurrence of LN-prRLN will bring increased risks to the parathyroid and recurrent laryngeal nerve. Therefore, it is necessary to accurately assess LN-prRLN for suitable surgical scope.

There are several limitations to our study. First, it comprised a single institutional retrospective analysis, and the findings may have limited generalizability to other populations of PTC patients. Additionally, selection bias existed in this study, as this was a retrospective study. Finally, this analysis was purely based on clinicopathological features, while considering imaging and molecule characteristics as study variables may strengthen the results (32).

In summary, in view of the rate of LN-prRLN metastasis, the difficulties and complications of reoperation, thorough central lymph node dissection should be considered. ETE, lateral lymph nodes metastasis, and LN-arRLN metastasis assessments are helpful in predicting the metastasis to LN-prRLN. A scoring system was also developed to identify which patients have a high risk of LN-prRLN metastasis and need complete resection. Even in the cN0 stage, LN-prRLN dissection should be performed once central lymph node metastasis is considered.

## Data Availability Statement

The original contributions presented in the study are included in the article/supplementary material. Further inquiries can be directed to the corresponding authors.

## Ethics Statement

The studies involving human participants were reviewed and approved by Ethics Committee of Tianjin Medical University Cancer Hospital. The patients/participants provided their written informed consent to participate in this study.

## Author Contributions

CJ, YSW, XDW contributed to conception and design of the study. MQZ and YSD organized the database. BY, HL, YW, PC and JJZ performed the statistical analysis. MQZ and YSD wrote the manuscript. All authors contributed to manuscript revision, read, and approved the submitted version.

## Conflict of Interest

The authors declare that the research was conducted in the absence of any commercial or financial relationships that could be construed as a potential conflict of interest.

## Publisher’s Note

All claims expressed in this article are solely those of the authors and do not necessarily represent those of their affiliated organizations, or those of the publisher, the editors and the reviewers. Any product that may be evaluated in this article, or claim that may be made by its manufacturer, is not guaranteed or endorsed by the publisher.
